# Supplementary figures and images for: In vivo osseointegration evaluation of implants coated with nanostructured hydroxyapatite in low density bone
Source: PLoS One. 2023 Feb 22;18(2):e0282067. doi: 10.1371/journal.pone.0282067 (PMC9946243; doi:10.1371/journal.pone.0282067)

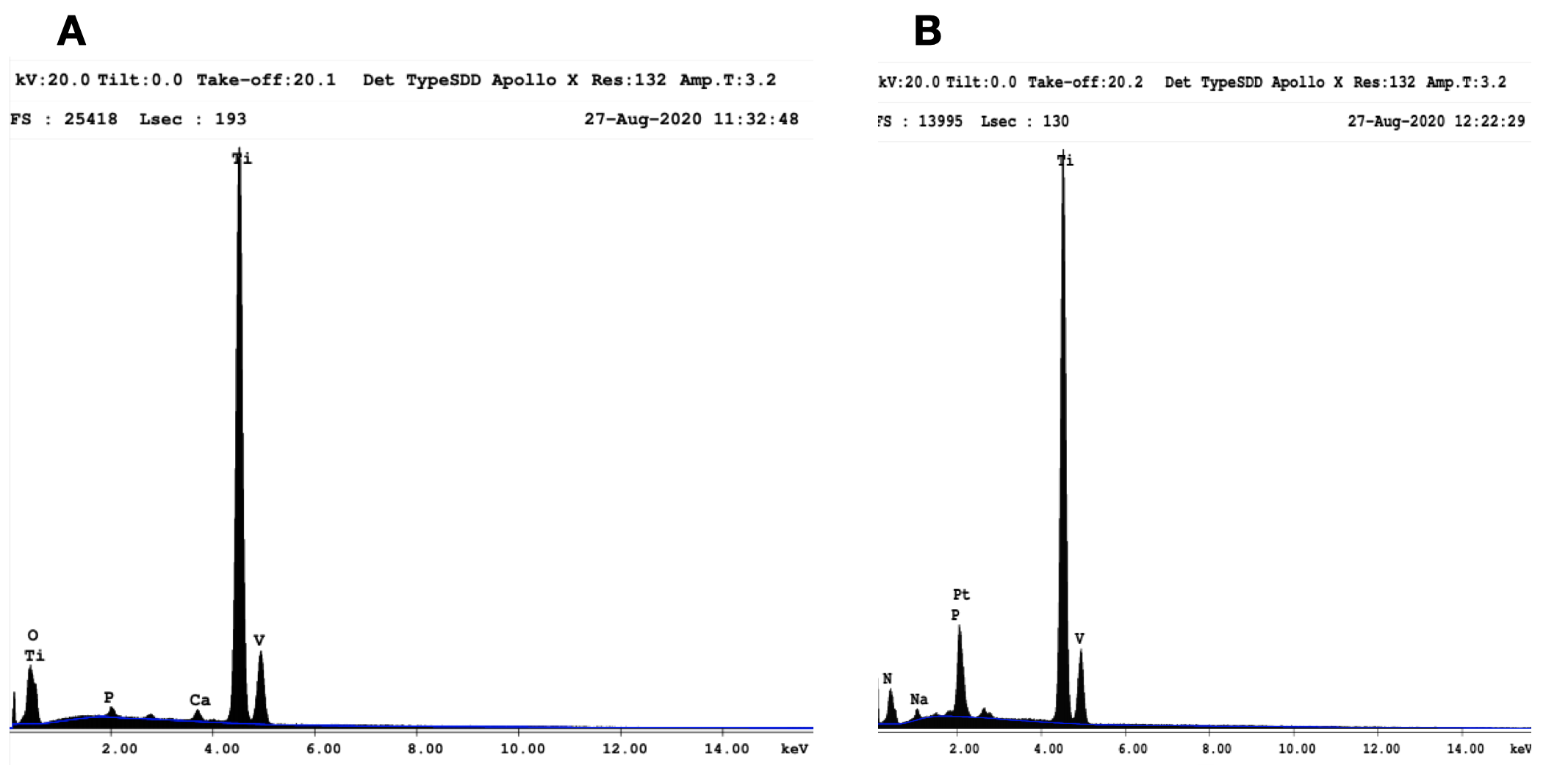

Supplement: S1 Fig — Peaks of titanium, vanadium, and phosphorus were observed in both groups. (TIF) [file pone.0282067.s001.tif]
