# Supplementary material for: In vivo osseointegration evaluation of implants coated with nanostructured hydroxyapatite in low density bone
Source: PLoS One. 2023 Feb 22;18(2):e0282067. doi: 10.1371/journal.pone.0282067 (PMC9946243; doi:10.1371/journal.pone.0282067)
Supplement: S1 Table — (DOCX) [file pone.0282067.s002.docx]

**Supplementary Table 1. Mean, Confidence interval, minimum and maximum values of bone area fraction occupancy (BAFo) and bone-implant contact (BIC) for DAA and HANano® groups after 21- and 28-days post-implantation.**

| Period |  |  |  | | **BAFo (%)** |  |  |  | Period |  |  | **BIC (%)** | | | |  |  |
| --- | --- | --- | --- | --- | --- | --- | --- | --- | --- | --- | --- | --- | --- | --- | --- | --- | --- |
| (Days) | Group | Mean (n=5) |  | CI | | Min | Max |  | (Days) | Group | Mean (n=5) | | |  | CI | Min | Max |
| **14** | DAA | 40.04 |  | 31.09 – 48.99 | | 30.91 | 47.19 |  | **14** | DAA | 56.76 |  | 40.22 – 73.31 | | | 37.22 | 72.76 |
|  | HANano® | 47.96 |  | 41.29 – 54.64 | | 43.12 | 57.14 |  |  | HANano® | 66.09 |  | 49.80 – 82.37 | | | 51.43 | 77.99 |
| **28** | DAA | 54.31 | ** | 50.18 – 58.45 | | 50.07 | 57.19 |  | **28** | DAA | 71.05 |  | 62.26 - 79.85 | | | 61.65 | 78.75 |
|  | HANano® | 65.53 | ** | 57.80 – 73.27 | | 59.69 | 72.75 |  |  | HANano® | 82.27 | * | 78.08 – 86.47 | | | 76.81 | 85.20 |

**Abbreviations**: BAFo (bone area fraction occupancy); BIC (bone-implant contact); Min (minimum); Max (maximum); CI (confidence interval at 95% of significance. (*****) Significant difference *vs*. the same group in the previous experimental period.
