# Supplementary material for: In vivo osseointegration evaluation of implants coated with nanostructured hydroxyapatite in low density bone
Source: PLoS One. 2023 Feb 22;18(2):e0282067. doi: 10.1371/journal.pone.0282067 (PMC9946243; doi:10.1371/journal.pone.0282067)
Supplement: S2 Table — (DOCX) [file pone.0282067.s003.docx]

**Supplementary table 2. Minimum, median and maximum values of insertion torque and resonance frequency for DAA and HANano® according to device/transducer.**

| Surfaces |  | **Insertion torque** | | |  | **Osstell/Smart Peg** | | |  | **Penguin/Multi Peg** | | |  |
| --- | --- | --- | --- | --- | --- | --- | --- | --- | --- | --- | --- | --- | --- |
|  |  | Minimum  (N/cm) | Median  (N/cm) | Maximum  (N/cm) |  | Minimum  (ISQ) | Median  (ISQ) | Maximum  (ISQ) |  | Minimum  (RFA) | Median  (RFA) | Maximum  (RFA) |  |
| **DAA** |  | 60 | 80 | 80 |  | 70 | 74 | 78 |  | 79 | 81 | 86 | * |
| **HAnano** |  | 60 | 70 | 80 |  | 69 | 75 | 76 |  | 76 | 82 | 82 | * |

**Abbreviations**: SD (standard deviation); ISQ (implant stability coefficient); RFA (resonance frequency analysis).

(*) Significant difference vs Osstell/Smart Peg.
