# Supplementary material for: In vivo osseointegration evaluation of implants coated with nanostructured hydroxyapatite in low density bone
Source: PLoS One. 2023 Feb 22;18(2):e0282067. doi: 10.1371/journal.pone.0282067 (PMC9946243; doi:10.1371/journal.pone.0282067)
Supplement: Attachment — Submitted filename: Response to Reviewers 2.docx [file pone.0282067.s004.docx]

**To: Academic Editor of PLOS ONE Wenguo Cui**

**Manuscript: PONE-D-22-24153**

**Title: *IN VIVO* OSSEOINTEGRATION EVALUATION OF IMPLANTS COATED WITH NANOSTRUCTURED HYDROXYAPATITE IN LOW DENSITY BONE**

Thank you for the attention you have given to our manuscript originally entitled **“*In vivo* osseointegration evaluation of implants coated with nanostructured hydroxyapatite in low density bone”.**

Here are the answers to the demands sent in the last mail:

“1. Thank you for updating your financial disclosure statement in the submission system. This is truly appreciated. However, please also note that you need to update your funding information section in the submission form to match the details included in the financial disclosure section.”

**Answer: Thanks for the guidance. Made the disclorure in the submission system.**

“2. Thank you for returning your revised manuscript. We note that there is still significant overlap with the following text:

<https://www.dovepress.com/the-influence-of-nanostructured-hydroxyapatite-surface-in-the-early-st-peer-reviewed-fulltext-article-IJN>

We would like to make you aware that copying extracts from previous publications, especially outside the methods section, word-for-word is unacceptable. In addition, the reproduction of text from published reports has implications for the copyright that may apply to the publications.

Please further address this overlap before we proceed.”

**Answer: The authors used the www.draftable.com platform to compare the texts and observed text similarities in the Conflict of Interest area and the legend of Figure 1. The authors altered similar texts; however, the bibliography references common between the two articles were not modified, remaining the same.**
